# Challenges and solutions to recruitment of neonates and children having cardiac surgery into a study using a novel sampling device

**DOI:** 10.1186/s13104-022-06088-y

**Published:** 2022-06-11

**Authors:** Terrie Walker-Smith, Daniel Fudulu, Aravind Ramesh, Karen Sheehan, Julie Madden, Lucy Culliford, Jonathan Evans, Gianni D. Angelini, Thomas Upton, Ben Gibbison

**Affiliations:** 1 Bristol Trials Centre, University of Bristol, Bristol, UK; 2 Bristol Heart Institute, University of Bristol, Bristol, UK; 3 University Hospitals Bristol and Weston NHS Foundation Trust, Bristol, UK; 4 Henry Wellcome LINE, University of Bristol, Bristol, UK; 5 Anaesthesia, Pain and Critical Care Sciences, University of Bristol, Bristol, UK

**Keywords:** Adrenal, Hypothalamic-pituitary-adrenal axis, Cardiac surgery, Paediatric endocrinology

## Abstract

**Objective:**

To narratively describe the challenges and solutions required in delivering a non-commercial study of children undergoing cardiac surgery using a novel subcutaneous hormone collection device.

**Results:**

The challenges faced by the research team are divided into those of conducting healthcare research in children and those specific to this study. Many of the issues of conducting healthcare research in children can and have been overcome by structural and institutional culture change–normalising and embedding research as part of good clinical care. The issues specific to insertion and maintenance of the novel collection device can be overcome by education and support of the clinical teams. The increased incentives and resources of commercial research may have overcome many of these.

## Introduction

Recruitment of patients to clinical research can often be problematic, regardless of whether the participants are children or adults [1]. In children, the recruitment issues are more complex due to the addition of a “third agent”—the parent or carer with responsibilities for making decisions for the child. This generates several obstacles (real and perceived) which must be overcome to ensure that studies are delivered. Novel devices and additional invasive research procedures potentially increase and enhance these obstacles. We narratively describe the challenges and solutions encountered during a study of 78 neonates and children undergoing subcutaneous hormone collection with a novel sample collection device during and after cardiac surgery and cardiology procedures.

## Main text

**Fig. 1:**
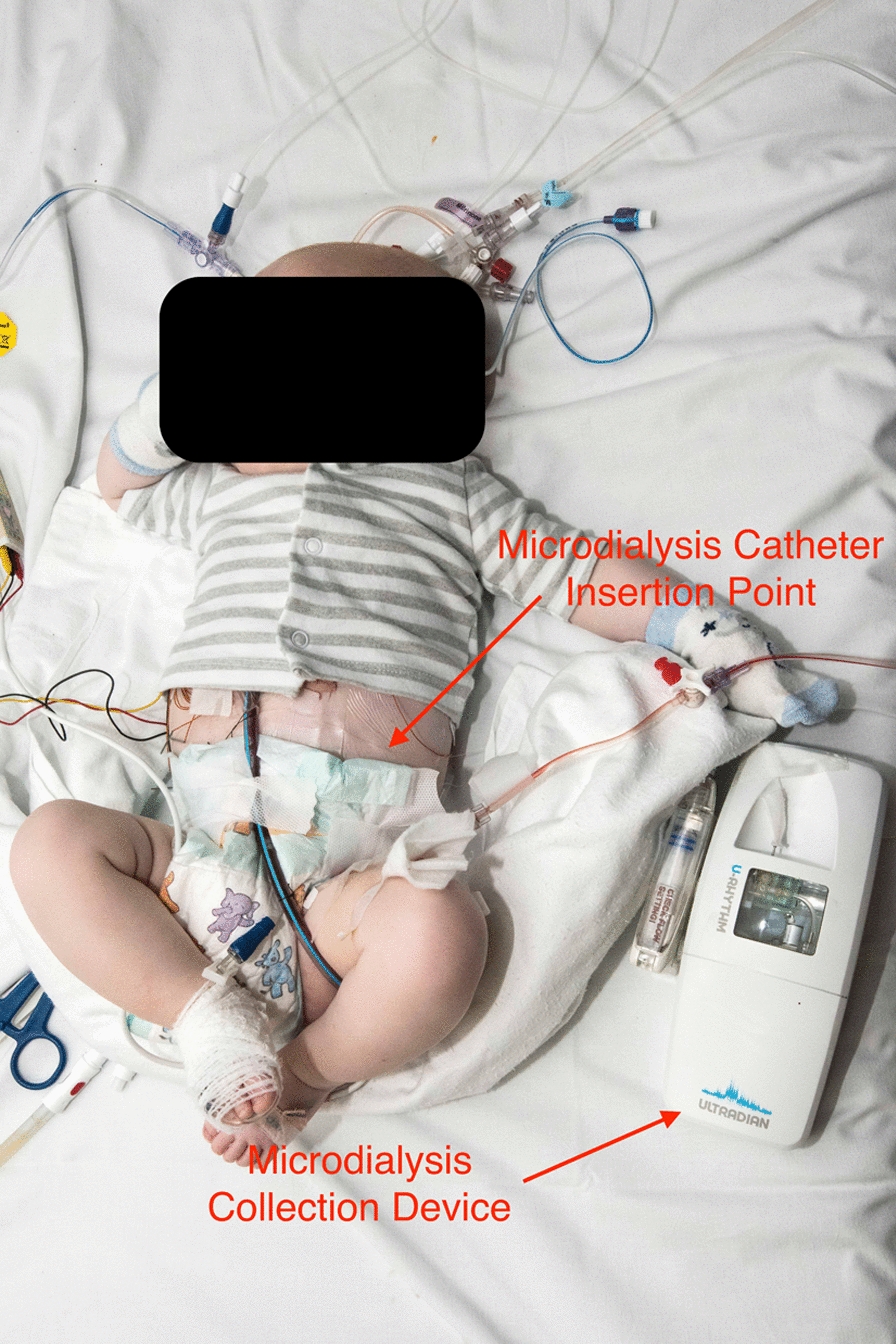
“The prototype microdialysis sample collector “U-RHYTHM” in use on a child who has recently undergone cardiac surgery. Fluid collected using a CE-marked clinical microdialysis probe (mDialysis, Sweden) is collected and automatically fractioned into samples which are stored in the U-RHYTHM device until the end of the sampling procedure, prior to retrieval and analysis (reproduced from [2])

The protocol has been reported previously [2], but in brief: The Peacock Study aims to characterise the hypothalamic-pituitary-adrenal axis of children during and after cardiac surgery. It is a prospective, two-centre, observational cohort study of 78 children (aged 0–16yrs), comparing those undergoing cardiac surgery with cardiopulmonary bypass and cardiology procedures (e.g. cardiac catheterisation) requiring a general anaesthetic. It takes place in 2 hospitals—one specialist paediatric hospital (treats only children, multiple conditions) and one specialist cardiothoracic hospital (treats only cardiothoracic conditions of both adults and children). The cohorts are grouped by age/cyanosis. A novel automated, subcutaneous microdialysis system [3] was used to collect samples for cortisol and cortisone every 20 min for 24-h. Prior to the Peacock study the system had never been trialled in neonates, infants or children. A microdialysis catheter is placed in the subcutaneous tissue of the child and connected to a pump that infuses an isotonic dialysis fluid, which equilibrates with the tissue fluid across the membrane. The fluid is collected by a fraction collector and the samples are stored within the device and decanted for assay using Liquid Chromatography–Mass Spectrometry. We also measured serum cortisol and adrenocorticotrophic hormone (ACTH) by taking blood from indwelling vascular catheters. Mathematical modelling is being used to build a model of the underlying adrenal control. A photograph of the device in use is in Fig. 1.

Estimated recruitment was a mean 3.5 patients per month for 3 years based on a 10% consent rate of an estimated 420 eligible patients per year (Total 1260). The study opened to recruitment on 14.7.2017 and closed on 31.12.21. Amendments to the protocol were approved on 7.10.2019 (to remove the acyanotic neonate group due to a paucity of ineligible patients) and 23.7.2020 to merge the pre/post-pubertal groups of the 10–16 years due to a paucity of pre-pubertal patients). The study recruited at a rate of 1.8 patients per month for the 53 months that the study was open. However, due to the restrictions because of the worldwide COVID-19 pandemic, the study was paused to recruitment for 3 months in 2020 and therefore the actual rate of recruitment was 2 patients per month. 1400 patients were screened across the two sites, the most common reasons for ineligibility were (i) Being of an age with an already complete cohort, (ii) recent use of corticosteroids and (iii) being in the cardiac catheter group with a planned discharge time of  ≤ 8 h post-procedure. The most common reasons for parents and patients declining participation were (i) feeling uneasy about the use of the novel device, (ii) did not want to participate in any research and (iii) parents too stressed to consider participation.

The challenges faced as part of this study can be divided into those of recruiting neonates and children into clinical studies and those specific to this study. The process for highlighting and resolving challenges was at regular study meetings (2–3 months), where the whole research team would meet (often online due to the multi-centre nature of the study). Recruitment, data completeness and safety were reviewed at every meeting. Solutions were discussed within the team. Minutes were taken of the meetings and actions to resolve problems were assigned to named individuals. The first agenda item of the following meeting was ensuring that actions from the previous meeting were complete. General issues of recruiting children are:

### Concern that it is not reasonable or appropriate to discuss participation in a study at a stressful time

Patients and families were happy to be approached about the study and many were keen to take part–something borne out by previous work [4]. In the year before the start of the COVID—19 pandemic, 870,000 people took part in health and social care research in England and around 10% of these were children [5]. This has increased substantially during and after the Covid-19 pandemic [5]. The Bristol site have a team of 3.4 Full Time Equivalent children’s cardiac surgery research nurses who screen, recruit and consent to observational and randomised studies [6]. The Bristol Royal Hospital for Children’s cardiac surgery research unit offers participation in research studies to every child undergoing cardiac surgery [7] with a consent rate of 84% (data to June 2021). Their familiarity with participant recruitment during the cardiac surgery process, alongside their education of clinical care staff regarding the benefits of clinical research, creates an environment where study and trial participation are normalised. Staff education regarding the benefits of research occurs at all levels of the organisation, from ward staff to Chief Executive Officer. This includes the direct financial incentives to the organisation for designing, co-ordinating and actively recruiting participants to research.

### Perception that paediatric research is “high-risk” and difficult

A “risk-based” approach to regulation and monitoring of clinical studies was incorporated in the International Council for Harmonisation of Technical Requirements for Pharmaceuticals for Human Use (ICH) GCP guidance in December 2016 [8]. This is because it was a non-interventional study, all consumables were CE marked, and sampling failure represented little risk to the child. The sponsor assessed this study to be 'low risk'. This designation facilitated risk-based reporting of safety events, protocol deviations and data monitoring. Appropriately minimising bureaucracy facilitates perceptions that study conduct is straightforward and increases staff time to concentrate on screening and recruitment. This study also requires comparatively little data collection and minimal study procedures for clinical staff—reducing the burden of work and giving them a positive impression of conducting research.

### Lack of knowledge and experience of undertaking paediatric research

This has been answered generally by the National Institute of Health Research (NIHR) in the UK and specifically within our unit through normalisation of participation in research. The NIHR has introduced GCP and specific “Informed Consent in Paediatric Research” e-learning packages that can be undertaken online by anyone within the UK NHS (https://learn.nihr.ac.uk). This builds on introductory GCP training taken by anyone designing or delivering research. Staff are often enthusiastic, and so it is our experience that the bureaucratic steps required to undertake paediatric research are often the barrier to undertaking paediatric research, rather than specifically gaining experience. This study in particular facilitated staff not previously involved in research to gain experience with minimal training requirements by implementing proportionate, rather than full GCP and protocol specific training.

## Challenges specific to this study are that

### Proposing the addition of an extra invasive procedure which confers no direct clinical benefit

Research nurses have a demonstration catheter that patients can see during early conversations about the study—allowing patients and their parents to see the size of the microdialysis collection tubing. Research Nurses also obtained permission from an early participant for a photograph to be used for recruitment purposes so that potential participants can see the catheter in situ. Real-world examples of the equipment both for participants to handle and visual examples of the device set-up during the first recruitment conversations help to allay fears regarding pain and discomfort from placement of the catheter and device.

### Recruitment of neonates requiring cardiac surgery can be difficult

Neonates often require surgery soon after delivery. Therefore, a multi-disciplinary approach is required between the cardiac research teams and the obstetric and neonatal clinical teams to highlight potential participants. Whilst families find this a stressful time; there is little evidence that they are displeased with being approached for research (consent rate for this study 64%). Lower consent rates in this group of patients does not mean that there is widespread unhappiness by parents at being approached for inclusion in research studies [9].

### Recruitment to age-specific cohorts.

This study recruited participants in age cohorts to define the changes that occurred across childhood. This requires increased research nurse time for screening to ensure that a patient was eligible for a particular cohort and that recruitment to a particular cohort was still available. A central screening file of all patients waiting for surgery means that individuals can be screened simultaneously for multiple studies, reducing time.

Difficult to recruit cohorts (e.g. acyanotic neonates) cause the recruitment time to be prolonged. This has an impact on study momentum as research teams feel that their initial success in recruitment then declines. This loss aversion [10] requires continuing support and encouragement from the study leadership team. When planning a study, it is also useful to ensure that there is a sufficient number of patients from these difficult to recruit cohorts that can be recruited within the life of the study and not just the overall numbers of patients recruited.

Criteria for entry into some cohorts needed to be changed. The paucity of acyanotic neonates undergoing cardiac surgery led us to remove this category and merge them into a single group of neonates. The interaction of sex steroids and corticosteroids initially led us to recruit two groups of 10–16-year-olds; one group was pre-pubertal and one group was post-pubertal. The median age for puberty has fallen over the last century [11, 12] (age 11 for females and 12 for males), and therefore the number of pre-pubertal patients of this age group presenting for surgery was negligible. We, therefore, also elected to merge this age cohort and record their pubertal status.

## There were some challenges specific to the device

### This was the first use of this device in children

Although well tested and validated in adults [13], the device had never been used in children and is not yet robust enough for routine clinical use. The clinical team could unwittingly disconnect the Prior to the Peacock study the system had never been trialled in neonates, infants or children sample collector from the sampling line during patient transfers. Furthermore, young and mobile participants (particularly age 1–5 years) would disconnect it themselves. This was remedied by research staff being present during known points of patient transfer [(e.g. between the operating room and Intensive Care Unit (ICU))]. Research staff would also educate clinical care staff about ensuring the integrity of the device/sampling line connection. With increased experience, both research and clinical staff found ways of securing the device connections and securing the sampling line to limit movement of the sampling lines (including revision of the original insertion technique by reinforcing connection points with silicon).

Some parents found the added responsibility of caring for the device as well as their ambulant, post-procedure child challenging and asked for the device to be disconnected early. The research team reassured parents from the point of recruitment that it was the research team responsibility to keep the device connected and ensure sampling. The improved packing and padding also de-medicalised the device and facilitated parental comfort with it.

### There was only a small number of people trained to insert the sample collector

There was a steep learning curve for the insertion of the device and a clear relationship between operator volume and sampling success rates. To ensure suitably trained staff were available to insert the device, we collated a pool of people, including research nurses and doctors to insert the device. Some staff were familiar with the device from other studies but had little experience in the paediatric (surgical) setting. They needed introducing to the paediatric environment and staff. Paediatric cardiac surgery/cardiology teams are usually small and are focussed on high-stakes outcomes. They can therefore be reluctant to have those who they do not know and, more importantly *trust* present. Humans display a preference for helping people whom they know and are familiar with [14], and therefore ensuring that research staff are “embedded” within clinical teams (even if they perform no clinical duties) plays one of the largest roles in facilitating research [15].

We also trained clinical staff who were interested in being part of the research, but who did not want, or have time to be part of the coordination and delivery of the study. This served two purposes: (1) It encourages people to become involved in research, and (2) Clinical staff felt “ownership” over the study and were willing to facilitate study conduct when they were performing their clinical duties.

### The time taken to insert the device before surgery prolonged the procedure time

Operating room efficiency is prioritised in many institutions. Insertion of the collection system takes 5–10 min and can delay the start of the operative procedure. This was linked to the reluctance of some clinicians to support the study. Steps were taken to ensure all stakeholders were aware of the study, its objectives, and its benefits (ethical and financial). The study team were present at operating room briefings and ensured participation was communicated in advance. They also minimised the time between patients on the operating list by facilitating the patient arriving in the procedure room in a timely manner. Equipment for insertion was set up in advance and in many cases could be done once the patient was anaesthetised and other clinical procedures were occurring.

## Limitations

The major limitation is that this report is based on the experiences of two UK centres conducting non-commercial research within a tertiary healthcare environment. Outside of this setting, some of these challenges may not be encountered or applicable. Commercial research generates more significant financial incentives for healthcare providers to participate in research and therefore more resources to deliver the research. Many of these challenges could be solved by having a full-time member of research staff available to insert and observe the device for the duration of the sampling.

The challenges reported were taken from feedback from research and clinical teams and were not the result of formal qualitative research sampling and analysis. Therefore, we cannot be sure that there is no bias—*confirmation* and *social acceptability* bias could persist. We may also not have captured the experiences of all stakeholders as we did not use a sampling framework. However, the challenges outlined are those which the teams have resolved and allowed the study to move to completion and therefore represent the most important.

## Data Availability

All data available on request to the corresponding author.

## Abbreviations

ACTH: Adreno-corticotrophic hormone
GCP: Good clinical practice
ICU: Intensive care unit
NIHR: National Institute of Health Research (UK)
