# Correction: Challenges and solutions to recruitment of neonates and children having cardiac surgery into a study using a novel sampling device

**DOI:** 10.1186/s13104-024-06729-4

**Published:** 2024-03-14

**Authors:** Terrie Walker‑Smith, Daniel Fudulu, Aravind Ramesh, Karen Sheehan, Julie Madden, Lucy Culliford, Jonathan Evans, Gianni D. Angelini, Thomas Upton, Ben Gibbison

**Affiliations:** 1 Bristol Trials Centre, University of Bristol, Bristol, UK; 2 University Hospitals Bristol and Weston NHS Foundation Trust, Bristol, UK; 3 University Hospitals Bristol and Weston NHS Foundation Trust, Bristol, UK; 4 Henry Wellcome LINE, University of Bristol, Bristol, UK; 5 Anaesthesia, Pain and Critical Care Sciences, University of Bristol, Bristol, UK

**BMC Research Notes (2022) 15:202**

10.1186/s13104-022-06088-y

Following the publication of the original article, we were informed that the British Heart Foundation funder grant number CH/1992027/7163 had been omitted from the funding information.

The original article has been revised.

